# Activity of telithromycin and comparators against bacterial pathogens isolated from 1,336 patients with clinically diagnosed acute sinusitis

**DOI:** 10.1186/1476-0711-3-15

**Published:** 2004-08-02

**Authors:** Joseph Dohar, Rafael Cantón, Robert Cohen, David John Farrell, David Felmingham

**Affiliations:** 1 Department of Pediatric Otolaryngology, Children's Hospital of Pittsburgh, Pittsburgh, USA; 2 Hospital Ramon y Cajal, Madrid, Spain; 3 Department of Microbiology, Intercommunal Hospital of Creteil, Creteil, France; 4 GR Micro Limited, London, United Kingdom

## Abstract

**Background:**

Increasing antimicrobial resistance among the key pathogens responsible for community-acquired respiratory tract infections has the potential to limit the effectiveness of antibiotics available to treat these infections. Since there are regional differences in the susceptibility patterns observed and treatment is frequently empirical, the selection of antibiotic therapy may be challenging. PROTEKT, a global, longitudinal multicentre surveillance study, tracks the activity of telithromycin and comparator antibacterial agents against key respiratory tract pathogens.

**Methods:**

In this analysis, we examine the prevalence of antibacterial resistance in 1,336 bacterial pathogens, isolated from adult and paediatric patients clinically diagnosed with acute bacterial sinusitis (ABS).

**Results and discussion:**

In total, 58.0%, 66.1%, and 55.8% of *S. pneumoniae *isolates were susceptible to penicillin, cefuroxime, and clarithromycin respectively. Combined macrolide resistance and reduced susceptibility to penicillin was present in 200/640 (31.3 %) of *S. pneumoniae *isolates (128 isolates were resistant to penicillin [MIC >= 2 mg/L], 72 intermediate [MIC 0.12–1 mg/L]) while 99.5% and 95.5% of isolates were susceptible to telithromycin and amoxicillin-clavulanate, respectively. In total, 88.2%, 87.5%, 99.4%, 100%, and 100% of *H. influenzae *isolates were susceptible to ampicillin, clarithromycin, cefuroxime, telithromycin, and amoxicillin-clavulanate, respectively. In vitro, telithromycin demonstrated the highest activity against *M. catarrhalis *(MIC_50 _= 0.06 mg/L, MIC_90 _= 0.12 mg/L).

**Conclusion:**

The high *in vitro *activity of against pathogens commonly isolated in ABS, together with a once daily dosing regimen and clinical efficacy with 5-day course of therapy, suggest that telithromycin may play a role in the empiric treatment of ABS.

## Introduction

The incidences of both the acute and chronic forms of sinusitis have been increasing, and between 10 and 15% of the population of central Europe are affected annually [1]. There are an estimated 30 million cases of ABS in the USA each year [2-4]. Acute sinusitis accounts for 0.5–2.0% of all upper respiratory tract infections in adults and between 5–10% in children and therefore is a common reason for visits to primary care physicians [5]. Although usually mild in severity, complications can be life threatening, including meningitis, brain abscess, orbital cellulitis and abscess, subempyema, osteomyelitis, and nasal polyposis [6-9].

*S. pneumoniae *is the most common pathogenic bacterium responsible for ABS, isolated in 30–50% of cases, followed by *H. influenzae*, isolated in 20–40% of cases. *Moraxella catarrhalis *is isolated in 5–10% of cases, beta haemolytic streptococci in less than 5%, and *Staphylococcus aureus *in less than 10% although it is often found co-infecting with other bacteria [10].

Treatment options for ABS are controversial as up to 40% of patients recover spontaneously, however, studies have shown that treatment with an antibacterial reduces the time to recovery from sinusitis, improves symptoms, and helps to prevent complications [11,12]. Guidelines on antibacterial use for ABS vary, possibly because of different regulations, antibacterial resistance patterns, and etiology in different countries, however, the choice of first-line antibacterial is similar across treatment guidelines [3,13-17]. Nearly all recommend amoxicillin, as it is active against the major causative pathogens of AMS and is generally well tolerated. For patients with penicillin allergy, the recommended first-line agents vary in different countries. Trimethoprim or trimethoprim-sulfamethoxazole is commonly recommended [3,14]. In addition, the French guidelines have recently been reviewed and telithromycin has been included as an alternative first-line agent [15]. Macrolides are not included in the French guidelines due to the high macrolide resistance prevalence in France [15,18].

Second line, or alternative antibacterial agents of choice, are clarithromycin or second-generation cephalosporins such as cefuroxime and cefpodoxime, third-generation cephalosporins such as cefdinir, and trimethoprim-sulphamethoxazole [3,5,16,17]. Telithromycin has been recommended as an alternative agent in Germany [13,16]. High-dose amoxicillin-clavulanate should be used if the patient does not improve [3,16,17]. In France, anti-pneumococcal fluoroquinolones are recommended after bacterial confirmation or if the patient is at high risk of complications [15]. A single dose of ceftriaxone can be used in a child who cannot be treated orally, i.e. vomiting [17].

However, due to increasing levels of resistance in bacterial respiratory tract pathogens to these commonly used antibacterials (particularly the rapid emergence of penicillin-and macrolide-resistant strains of pneumococci), new agents are required that have high *in vitro *activity and demonstrated clinical efficacy against bacterial pathogens causing community-acquired respiratory tract infections (RTI's) [19-22]. Telithromycin is the first ketolide approved for clinical use. The ketolides are semisynthetic derivatives of the 14-membered ring macrolide erythromycin and have high *in vitro *activity against the common community-acquired RTI pathogens [23]. Clinical trials have demonstrated the efficacy and tolerability of telithromycin therapy in ABS [24-26].

The PROTEKT (Prospective Resistant Organism Tracking and Epidemiology for the Ketolide Telithromycin) study is a longitudinal, global multicentre surveillance study designed in part to determine the activity of telithromycin against community-acquired RTI isolates, in relation to the frequency of prescribing, in the regions where the study is conducted [27]. The aim of this paper is to focus on the data gathered in the PROTEKT surveillance study to determine the in vitro efficacy of the new ketolide telithromycin and comparator agents against bacterial pathogens isolated from the subset of patients with clinically diagnosed ABS collected in PROTEKT (2000–2001, and 2001–2002).

## Materials and Methods

### Patients and bacterial isolates

Detailed study design, including patient selection and methodology for isolate identification and storage in the PROTEKT study has been described previously [27]. The isolates in this sub-study of PROTEKT were selected from those patients presenting with clinically diagnosed ABS in which the isolates were determined clinically to be the pathogenic organism and the specimen type was sinus aspirate or nasopharyngeal swab/aspirate only. Methodology for sinus aspiration was that used routinely by the investigator.

### Antimicrobial testing

MIC susceptibility status was determined, using the National Committee of Clinical and Laboratory Standards (NCCLS) breakpoints, at a central laboratory (GR Micro Ltd, London, UK) from a panel of existing and new antibacterials, using the NCCLS broth microdilution method and lyophilised microtitre plates (Sensititre, Trek Diagnostics) [28]. NCCLS breakpoints were used for interpretation of MIC's [29]. Tentative NCCLS breakpoints for telithromycin are: *S. pneumoniae *and *S. aureus*, ≤ 1 μg/ml is susceptible, 2 μg/ml is intermediate, and ≥ 4 μg/ml is resistant; for *Haemophilus influenzae*, ≤ 4 μg/ml is susceptible, 8 μg/ml is intermediate, and ≥ 16 μg/ml is resistant [29].

### Statistical analysis

Statistical analysis was performed using a χ^2 ^test.

## Results

A total of 1,336 bacterial pathogens in all were collected from 25 countries within Western Europe (*n *= 652), North America (*n *= 14), Latin America (*n *= 207), Asia (*n *= 464), Eastern Europe (*n *= 68), Australia (*n *= 2), and South Africa (*n *= 126) in the PROTEKT study from years 2000–2001 and 2001–2002 for analysis of the susceptibility of bacterial pathogens isolated from patients with acute sinusitis. Gender distribution was 52.2% male (695 patients), 46.7% female (624 patients); gender was not provided for 1.3% of patients. Almost two thirds (66.3%) of patients were in the 0–12 year age group, one third (29.7%) in the 13–65 year age group, 2.7% in the over 65 year age group and age was not specified in 1.3% of patients. *S. pneumoniae *was the pathogen most frequently isolated (47.9% of isolates) followed by *H. influenzae *(24.6% of isolates) (Table 1).

**Table 1:** Distribution of species by specimen type for the 1336 bacterial pathogens causing acute sinusitis [n (%)]

**Specimen**	***S. pneumoniae***	***H. influenzae***	***M. catarrhalis***	***S. aureus***	***S. pyogenes***	**Total**
Sinus	272 (47.5)	148 (25.9)	67 (11.7)	64 (11.2)	21 (3.7)	572 (42.8)
Nasopharynx^1^	368 (48.1)	181 (23.7)	145 (19.0)	52 (6.8)	18 (2.4)	764 (57.2)
Total	640 (47.9)	329 (24.6)	212 (15.9)	116 (8.7)	39 (2.9)	1336 (100)

^1^Aspirate or swab

MIC data for isolates from patients with ABS demonstrated that the *in vitro *activity of telithromycin against gram-positive cocci was similar to amoxicillin-clavulanate and was higher and more potent than clarithromycin and beta-lactams tested such as cefuroxime (Table 2). In total, 99.5 % of streptococcal isolates were susceptible to telithromycin. With the exception of *S. aureus *isolates more than 90% of gram-positive cocci were inhibited at a telithromycin MIC of 0.25 mg/L (Table 2).

**Table 2:** *In vitro *activity of antibacterial agents and percent susceptible against 1336 bacterial pathogens isolated from patients with clinically diagnosed acute sinusitis

**Organism**	**N (total, SA^1^, NP^2^)**	**Antibiotic**	**MIC (mg/L)**			**Percent susceptible **(Total, *SA, NP*)
				
			**Range**	**50**	**90**	
***S. pneumoniae***	640, 272, 368	Penicillin	0.008 – 8	0.06	2	58.0, *64.7, 53.0*
		Amoxicillin-clavulanate	0.015 – 8	0.03	2	95.5, *95.2, 95.7*
		Cefuroxime	0.015 – 16	0.12	8	66.1, *73.2, 60.9*
		Cefpodoxime	0.12 – 32	0.12	2	65.0, *71.7, 60.1*
		Trimethoprim-sulphamethoxazole	0.12 – 32	0.5	8	56.3, *58.1, 54.9*
		Erythromycin	0.03 - >64	0.06	>64	55.9, *60.7, 52.5*
		Clarithromycin	0.015 - >32	0.06	>32	55.8, *60.7, 52.2*
		Azithromycin	0.03 - >64	0.12	>64	55.8, *60.7, 52.2*
		Telithromycin	0.008 – 8	0.015	0.12	99.5, *98.9, 100*
***H. influenzae***	329, 148, 181	Ampicillin	0.12 – 32	0.25	8	88.2, *91.2, 85.6*
		Amoxicillin-clavulanate	0.12 – 4	0.5	1	100, 100,100
		Cefuroxime	0.12 – 16	1	2	99.4, *99.3, 99.5*
		Cefpodoxime	0.015 – 4	0.06	0.25	99.4, *100, 98.9*
		Cefdinir	0.06 – 4	0.25	0.5	97.3, 96.0, 98.3
		Trimethoprim-sulphamethoxazole	0.03 – 16	0.06	4	84.5, *82.4, 86.2*
		Erythromycin	0.25 – 16	4	8	-^3^
		Clarithromycin	0.25 – 32	8	16	87.5, *87.2, 87.9*
		Azithromycin	0.06 – 4	1	2	100, 100,100
		Telithromycin	0.06 – 4	1	2	100, 100,100
***M. catarrhalis***	212, 67, 145	Ampicillin	0.12 – 32	4	16	-
		Amoxicillin-clavulanate	0.12 – 0.5	0.12	0.25	-
		Cefuroxime	0.12 – 16	1	4	-
		Cefpodoxime	0.06 – 4	0.5	1	-
		Cefdinir	0.06 – 1	0.12	0.25	-
		Trimethoprim-sulphamethoxazole	0.03 – 2	0.12	0.25	-
		Erythromycin	0.25 – 1	0.25	0.25	-
		Clarithromycin	0.25 – 0.5	0.25	0.25	-
		Azithromycin	0.06 – 0.25	0.06	0.06	-
		Telithromycin	0.004 – 0.5	0.06	0.12	-
***S. aureus***	116, 64, 52	Methicillin	-	-	-	90.5, *92.2, 88.5*
		Amoxicillin-clavulanate	0.06 – 8	0.5	4	90.5, *92.2, 88.5*
		Cefuroxime	0.5 – 16	1	2	90.5, *92.2, 88.5*
		Cefpodoxime	1 – 32	2	4	88.8, *92.2, 84.6*
		Trimethoprim-sulphamethoxazole	0.12 – 32	0.12	0.12	96.6, *95.3, 98.1*
		Erythromycin	0.03 - >64	0.25	>64	69.0, *73.4, 63.5*
		Clarithromycin	0.015 - >32	0.25	>32	69.8, *75.0, 63.5*
		Azithromycin	0.03 - >64	0.5	>64	69.0, *75.0, 61.5*
		Telithromycin	0.015 - >32	0.06	2	89.7, *89.1, 90.4*
***S. pyogenes***	39, 21, 18	Penicillin	0.008 – 0.008	0.008	0.008	100, *100,100*
		Amoxicillin-clavulanate	0.008 – 0.03	0.015	0.015	100, *100,100*
		Cefuroxime	0.015 – 0.015	0.015	0.015	100, *100,100*
		Cefpodoxime	0.12 – 0.12	0.12	0.12	100, *100,100*
		Trimethoprim-sulphamethoxazole	0.12 – 0.5	0.12	0.25	-
		Erythromycin	0.03 – 4	0.06	0.25	92.3, *100, 83.3*
		Clarithromycin	0.015 – 2	0.03	0.25	92.3, *100, 83.3*
		Azithromycin	0.03 – 16	0.12	0.25	92.3, *100, 83.3*
		Telithromycin	0.008 – 0.12	0.015	0.015	-

^1^Sinus aspirate ^2^Nasopharyngeal aspirate or swab ^3^No NCCLS interpretive guidelines available or pending

Resistance to most antibiotics was slightly greater in nasopharyngeal specimens than sinus aspirates (Table 2). Considerable variation in *in vitro *antibiotic activity was apparent between geographical regions as observed in the key examples shown in Table 3. Insufficient data were available for analysis by country.

**Table 3:** Key example of regional variation in *in vitro *antibiotic activity

	***Streptococcus pneumoniae***			***Haemophilus influenzae***		
REGION^1^	N	Penicillin susceptible	Erythromycin susceptible	N	Beta-lactamase positive

Eastern Europe	40	70.0%	82.5%	13	0.0%
Far East	185	24.9%	15.7%	96	13.5%
Latin America	108	63.0%	73.1%	31	6.5%
South Africa	61	37.7%	59.0%	34	2.9%
Western Europe	246	83.7%	73.6%	153	11.8%
Grand Total	640	58.0%	55.9%	329	10.3%

^1^Australasia and North America not included due to insufficient data

Combined macrolide resistance and reduced susceptibility to penicillin was present in 200/640 (31.3 %) of *S. pneumoniae *isolates (128 isolates were resistant to penicillin [MIC >= 2 mg/L], 72 intermediate [MIC 0.12–1 mg/L]). Of note, 3 isolates of *S. pneumoniae *were non-susceptible to telithromycin (2 isolates intermediate with an MIC of 2 mg/L, 1 isolate resistant with an MIC of 8 mg/L). This represented 0.5% of isolates, a value that is significantly (p < 0.001) lower than those obtained by erythromycin (44.1%), clarithromycin (44.2%) and cefuroxime (33.9%).

Of the 329 *H. influenzae *isolates, 34 (10.3 %) were positive for β-lactamase production. All isolates of *H. influenzae *were susceptible to amoxycillin-clavulanate and telithromycin with an MIC_90 _of 1 and 2 mg/L, respectively. Amoxycillin-clavulanate and telithromycin were more potent and had greater activity than clarithromycin (MIC_90 _= 16 mg/L, 87.5% susceptible). This activity was comparable to azithromycin (MIC_90 _= 2 mg/l, 100% susceptible).

Although the number of *S. aureus *isolated from the total number of specimens was small (116/1366 isolates), telithromycin was as efficacious as comparators. Of the 116 isolates, 11 were resistant to methicillin (MRSA) and 105 were methicillin susceptible (MSSA). Ninety-nine (94.3%) of the MSSA isolates and 5 of the 11 MRSA isolates were susceptible to telithromycin. Of note, all of the *S. pyogenes *isolates were inhibited by ≤ 1 mg/L telithromycin, despite 17.7% resistance to erythromycin and clarithromycin.

Telithromycin was the most potent antimicrobial against *M. catarrhalis *with an MIC_50 _of 0.06 mg/L and MIC_90 _of 0.12 mg/L. β-lactamase production was detected in 97.6% of these isolates.

## Discussion

The data in this analysis demonstrates that telithromycin has high *in vitro *activity against bacterial pathogens isolated from a large, globally distributed population of patients diagnosed with ABS. Telithromycin was the most active and potent agent against all isolates of the pathogens isolated from patients with ABS with 99.4% of isolates susceptible. Not surprisingly, high levels of penicillin resistance, macrolide resistance, and combined penicillin and macrolides resistance were prevalent in *S. pneumoniae *although prevalence varied widely between geographical regions.

Amoxicillin has been the treatment of choice in ABS because of its general effectiveness, safety, tolerability, low cost and narrow spectrum [17]. The high prevalence of beta-lactamase in *H. influenzae *and *M. catarrhalis *found in the present study demonstrate compromised *in vitro *efficacy of amoxicillin against these isolates. Although the cephalosporins (cefuroxime, cefpodoxime and cefdinir) showed high activity against *H. influenzae *(including beta-lactamase positive strains), resistance to these agents was high in *S. pneumoniae*: >30% for cefuroxime and cefpodoxime – cefdinir was not tested against pneumococci in PROTEKT, however susceptibility is usually similar to the other cephalosporins reported here [30].

Similarly, macrolides are prescribed in various countries for ABS and an overall resistance rate for *S. pneumoniae *of 44.1% to erythromycin, azithromycin, and clarithromycin was found. Trimethoprim-sulfamethoxazole activity was low for *S. pneumoniae *(56.3% susceptible) and decreased for *H. influenzae *(84.5% susceptible).

Respiratory fluoroquinolones are recommended second-line treatment options in some countries (references needed to support this statement). However, recent evidence suggests that resistance to fluoroquinolones is rapidly developing in pneumococci and other pathogens (including gram-positive and gram-negative [31-34]. To preserve the long-term utility of fluoroquinolones, including their use in the treatment of serious non-respiratory infections, it has been recommended that respiratory fluoroquinolones be reserved for treating severe (e.g. hospitalized) community-acquired RTIs only [35,36].

The high prevalence of beta-lactam, macrolide, TMP-SMX resistance demonstrated in the large number of isolates from patients with clinically diagnosed sinusitis in our study demonstrates the need to be exploring new therapeutic options, especially in geographical regions of high prevalence such as the Far East.

The high *in vitro *activity of telithromycin against ABS pathogens reported in this study, regardeless of geographical region, also demonstrates its potential as an empiric therapeutic option for ABS. There are several other reasons to consider this option – 1) High rates of clinical cure and bacteriological eradication have been demonstrated using telithromycin against sinus isolates of *S. pneumoniae*, *H. influenzae*, *M. catarrhalis *and *S. aureus *[24-26]. 2) Telithromycin has been shown to have a targeted spectrum of activity against the major bacterial respiratory tract pathogens and has less effect on normal bacterial ecology [37-39]. 3) The pharmacokinetic profile of oral telithromycin allows it to be prescribed with a dosing regime of 800 mg once daily for 5 days [40,41]. This contrasts favourably with its comparators, where a 10 – 14 day course with administration either 2 or 3 times daily, depending on the chosen antibacterial, is generally prescribed. Studies have shown that the once daily dosing regime affords greater patient treatment compliance, thereby avoiding clinical failure and the ensuing development of antibacterial resistance [41-43]. 5) Telithromycin has been shown to have high penetration levels in paranasal sinuses, and it is preferentially absorbed by polymorphonuclear neutrophils (PMNs) within the azurophil granules allowing effective delivery to phagocytized intracellular bacteria [44,45].

Although this study provides valuable information on the overall antimicrobial profile of bacteria causing ABS care should be taken when interpreting data related to specific demographics. A major limitation of this study, inherent to most surveillance studies, is the requirement for collecting centers to fulfill a specified quota of isolates over a defined time period (1 year). If, for instance, 1 center manages to fulfill their quota for *S. pneumoniae *isolates from patients with community-acquired pneumonia, they may then only send *H. influenzae *from patients with ABS to fulfill their quota for this organism. Thus, the potential exists to over or under estimate the prevalence of a species in a particular disease. A further limitation of this study is it is restricted to the major bacterial pathogens causing sinusitis and does not therefore assess anaerobic bacteria, which are also known to be involved in this disease. However, a recent study of sinus puncture specimens demonstrated that telithromycin had good *in vitro *activity against anaerobes involved in sinusitis [46].

The inclusion of nasopharyngeal specimens is a potential limitation of this study and the higher rate of resistance compared to sinus aspirates may indicate some isolates were nasopharyngeal flora rather than pathogens. However, the difference in resistance prevalence between nasopharyngeal specimens and sinus aspirates was not great for any species/antibiotic combination, and assuming the majority of isolates were the responsible pathogen, significant bias of resistance patterns is unlikely. The treatment of ABS is complicated by a difficulty in establishing the causative pathogen(s). Sampling of infected fluid using sinus puncture is a painful and rare procedure [47]. Nasopharyngeal culture is a painless and reliable method that can help identify patients that may benefit from antibacterial therapy [48] and hence, could be useful in determining antibiotic resistance implicated in sinusitis – particularly *Streptococcus pneumoniae *and *Haemophilus influenzae*. Additionally, there are regional differences in the susceptibility patterns observed and, as therapy is usually empirical, choosing an effective therapy can be challenging [18,20,49].

In summary, the data presented here demonstrates that telithromycin has good *in vitro *activity against *S. pneumoniae*, *H. influenzae, M. catarrhalis *and *S. aureus *respiratory pathogens commonly isolated in ABS. It is as active as or more active than antibacterial agents that are currently used in this clinical setting. The development of resistance will always be a threat to the usefulness of antibacterial compounds, however surveillance studies such as PROTEKT allow the rapid detection and characterization of resistance mechanisms and highlight the need for and examine the *in vitro *efficacy of newer antibacterial agents. Providing careful surveillance for the development of resistance is maintained telithromycin currently offers a useful therapeutic option in the treatment of AS.

## Authors' contributions

JD, RCa and RCo reviewed the data and provided clinical and microbiological interpretation and discussion. DF and DJF participated in the design of the study, supervised the scientific testing, and provided data analysis, microbiological interpretation and discussion. All authors drafted the manuscript. All authors read and approved the final manuscript.
